# The Long-Term Impact of Postoperative Educational Programs on Weight Loss After Roux-en-Y Gastric Bypass

**DOI:** 10.1007/s11695-022-06187-6

**Published:** 2022-07-06

**Authors:** Kirsti K. Bjerkan, Jorunn Sandvik, Siren Nymo, Hallvard Græslie, Gjermund Johnsen, Ronald Mårvik, Åsne A. Hyldmo, Bård Eirik Kulseng, Kjetil Laurits Høydal, Dag Arne L. Hoff

**Affiliations:** 1 Faculty of Social Science and History, Volda University College, Joplassvegen 1, 6103 Volda, Norway; 2 Department of Surgery, Møre and Romsdal Hospital Trust, Ålesund Hospital, Ålesund, Norway; 3 Centre for Obesity and Innovation (ObeCe), Clinic of Surgery, St. Olav University Hospital, Trondheim, Norway; 4 Obesity Research Group, Department of Clinical and Molecular Medicine, Faculty of Medicine and Health Sciences, Norwegian University of Science and Technology (NTNU), Trondheim, Norway; 5 Nord-Trøndelag Hospital Trust, Clinic of Surgery, Namsos Hospital, Namsos, Norway; 6 Norwegian National Advisory Unit On Advanced Laparoscopic Surgery, Clinic of Surgery, St. Olav’s University Hospital, Trondheim, Norway; 7 Department of Physical Education, Volda University College, Volda, Norway; 8 Department of Research and Innovation, Møre and Romsdal Hospital Trust, Ålesund, Norway; 9 Department of Clinical and Molecular Medicine, Faculty of Medicine and Health Sciences, NTNU, Trondheim, Norway

**Keywords:** Gastric bypass, Educational program, Group session, Weight regain

## Abstract

**Purpose:**

Roux-en-Y gastric bypass (RYGB) is a well-documented treatment of severe obesity. Attending postoperative educational programs may improve the outcome. The aim of this study was to evaluate whether participation in educational programs lasting 2–3 years after RYGB influences long-term weight loss, weight regain, physical activity, and compliance to multivitamin supplements.

**Materials and Methods:**

The Bariatric Surgery Observation Study (BAROBS) is a multicenter retrospective, cross-sectional study 10–15 years after primary RYGB. Four hundred and ninety-seven participants answered questions regarding participation in postoperative educational programs. Participants were divided into frequent attendees (FA) and infrequent attendees (IFA) at the educational programs.

**Results:**

Ten to 15 years after surgery, a total weight loss (TWL) of 23.2 ± 11.6% were seen in the FA group vs 19.5 ± 12.6% in the IFA group, *p* < 0.001. Percent excess weight loss (%EWL) was 55.7 ± 28.9% vs 46.0 ± 31.1%, *p* < 0.001. Weight regain in percent of maximal weight loss for the FA was 32.1 ± 32.8% vs IFA 38.4 ± 40.0%, *p* = 0.052. No difference between the groups in compliance to multivitamin and physical activity.

**Conclusion:**

Participants with frequent participation in group-based educational programs had better weight loss outcomes 10–15 years after RYGB and tended to have less weight regain. There was no difference between the two groups in participants compliance to recommended multivitamin supplements and physical activity.

**Graphical abstract:**

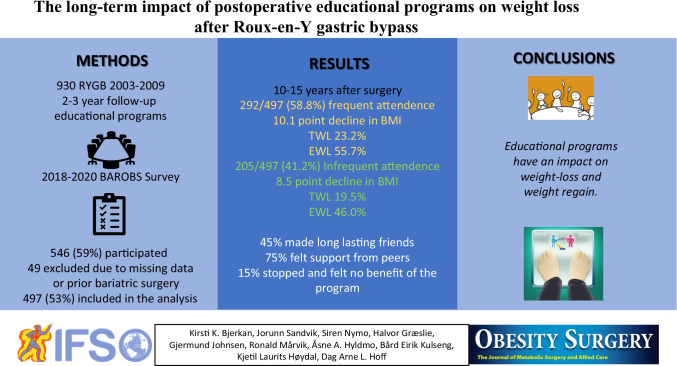

## Introduction

Obesity is a progressive and relapsing chronic disease [1]. Even though obesity surgery (OS) is the best-documented treatment for severe obesity, it is not a cure, as weight regain (WR) is common [2]. A systematic review of 18 reports found a weighted mean of 56.7%EWL 10 or more years after Roux-en-Y gastric bypass (RYGB) [3]. About one in four of the patients undergoing RYGB procedure in the Longitudinal Assessment of Bariatric Surgery (LABS) study did not achieve 20% weight reduction from operation to 7-year follow-up [4]. International guidelines recommend participation in postoperative support groups to optimize weight management after OS [5, 6]. However, knowledge about the long-term effect of education programs after OS is lacking and drop-out rates are high [6–8].

The World Health Organization (WHO) describes health literacy as “By improving people’s access to health information, and their capacity to use it effectively, health literacy is critical to empowerment” [9]. Patients tend to forget preoperative information after OS [10]. The postoperative education program repeats preoperative information and allows the patients to ask new questions. Patient education can be given individually in person or on a digital platform, or to a group of patients led by health professionals. It is important to identify predictors of successful weight loss and psychosocial outcomes to reduce patient distress and disappointment [11]. This paper aims to explore the long-term outcome of participation in group-based educational programs lasting 2 to 3 years postoperatively. We explore data on weight, self-rated health, adherence to recommended vitamin supplements, and physical activity 10–15 years after RYGB.

## Materials and Methods

**Fig. 1:**
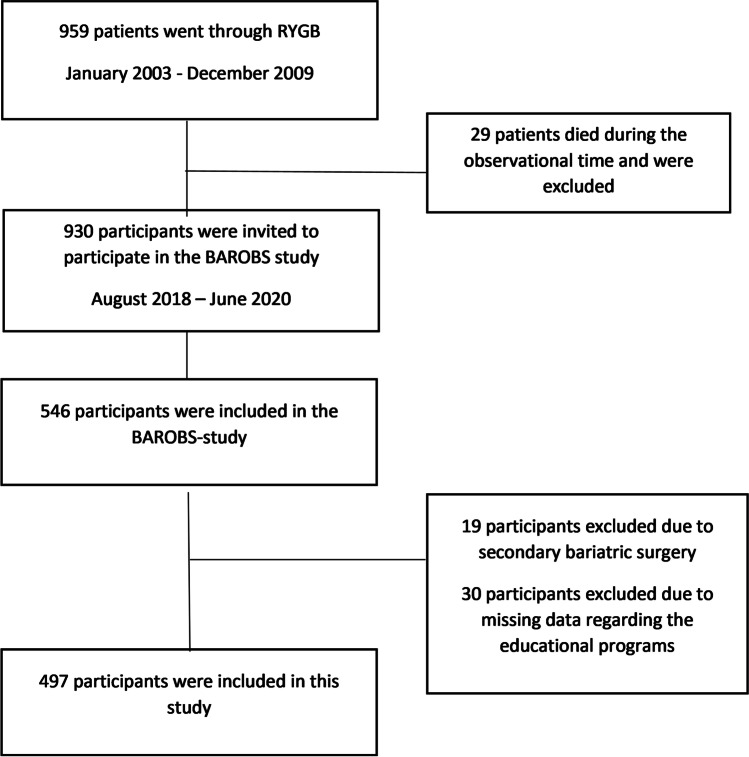
Participant flow chart. RYGB, Roux-en-Y gastric bypass

The Bariatric Surgery Observation Study (BAROBS) is a retrospective, multicenter, cross-sectional study exploring long-term effects after RYGB performed at three public hospitals in Central Norway Health Region between 2003 and 2009. Ten to 15 years after surgery, 546 out of 930 invited patients accepted to take part in the study. The BAROBS was approved by the local ethics committee (REK 2017/1828–21). BAROBS included a general health evaluation with clinical exams, blood tests, and a survey covering questions regarding lifestyle, social, physical, and mental health topics. The participants were also asked about their experiences with the educational programs and the hospital’s follow-up. Additional data was collected from the participants medical records. We made 49 exclusions in this paper due to either revisional procedures (*n* = 19) or missing data (*n* = 30) regarding the educational programs (Fig. 1). There were available data on 497 patients’ attendance in group-based educational programs. The RYGB procedure was standardized and performed laparoscopically according to the Lönroth technique, with an antegastric alimentary limb of 100 or 150 cm, depending on BMI below or above 50 kg/m^2^ [12].

A group-based educational program lasting 2 to 3 years after surgery was offered to all patients after the RYGB procedure. The purpose of the educational programs was to prepare the patients for life after surgery and empower the participants by searching for their motivation and skills to handle the lifestyle changes and thereby tackle psychological challenges. The groups were led by a clinical dietitian and a nurse at all three hospitals. However, there were minor differences in the curriculum, and the number of half-day sessions differed from five or ten within 2 or 3 years. Teaching sessions included the necessity of lifelong adherence to multivitamin-mineral supplements (MVS), healthy food choices, and physical activity. The sessions shifted from teaching through motivational technics and discussions around the table focusing on self-efficacy, expectations, experiences, and daily challenges. In addition, physiotherapists and psychologists attended some of the sessions. The educational programs were optional, but strongly recommended by members of the bariatric team in all three hospitals.

Weight and BMI were registered at the time of surgery, at the time of lowest registered weight (nadir) within the second year after surgery, and after 10–15 years. Weight changes were calculated as a percentage of total weight loss (%TWL), excess weight loss (%EWL), and ∆BMI [13]. WR was calculated in percentage of maximum weight loss according to King et al. who suggest classifying WR > 20% WR as weight regainers [14].

Participants who answered “they attended the group sessions as often as possible” in the questionnaire were frequent attendees (FA). The infrequent attendees (IFA) answered, “they attended sometimes or never.” The level of education is divided into two categories ≤ 12 years and > 12 years of school. Family income is divided into two categories, with a cut-off at 75.000 Euro/year, which is the average Norwegian household income. Our definition of “active > 150 min per week (min/week)” were participants who answered the questions “being active 30–60 min almost every day” or “active > 60 min at least 2–3 days or almost every day.” Intensity was categorized as low and high. Participants defined as compliant with MVS answered taking one from a selection of different supplements. Self-rated health was registered from an answer to the question: “In general, would you say your health is (1) excellent, (2) very good, (3) good, (4) fair or (5) poor.” The categories excellent, very good, and good are merged to “good,” and fair and poor is merged to “poor.”

### Statistical Analyses

Continuous variables were normally distributed and are reported as means with standard deviation (SD). An independent *t*-test is used to compare continuous normally distributed variables. The Pearson *χ*^2^ was used to compare categorical variables, and *p*-values were corrected for multiple hypothesis testing. Two-way ANOVA tests differences between groups and within the three hospitals. *p*-values of < 0.05 are considered significant.

The denominator will sometimes change in the results because there might be an uneven number of patients answering some of the questions. The data analyses were done with IBM SPSS statistics version 27 (SPSS Inc., Chicago, IL, USA) software. Graphs are made in GraphPad Prism version 9 (GraphPad Software, LLC, CA, USA).

## Results

At baseline, the 497 participants were 39.8 ± 8.8 years old, and the BMI was 44.4 ± 5.4 kg/m^2^. The mean time to follow-up was 140.8 ± 19.1 months (11.7 years). Two-hundred and ninety-two (58.8%) participants were categorized as FA in the educational program, and 241 (82.5%) of them were women. Among the 205/497 (41.2%) participants categorized as IFA, 154 (75.1%) were women. The FA were older than the IFA, respectively, 40.8 ± 8.9 years vs 38.4 ± 8.6 years (*p* = 0.003).

**Fig. 2:**
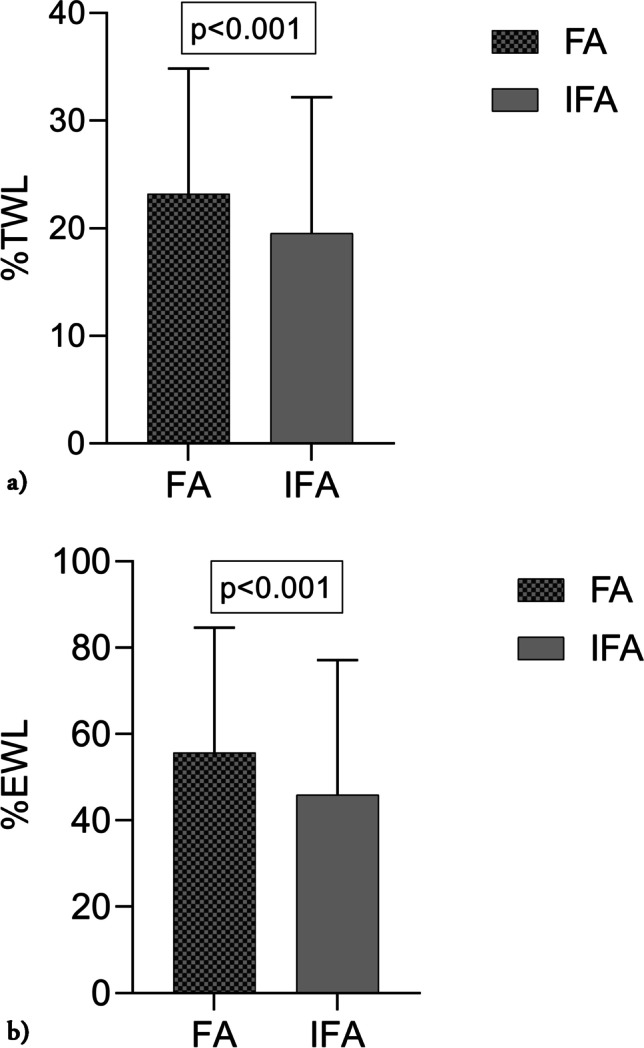
**a** %Total weight loss (%TWL) 12 years after surgery in frequent attendees (FA) and infrequent attendees (IFA) at the educational programs at the three hospitals. Results presented as mean ± SD, *p*-value in box above. **b** %Excess weight loss (%EWL) 12 years after surgery in frequent attendees (FA) and infrequent attendees (IFA) at the educational programs at the three hospitals. Results presented as mean ± SD, *p*-value in box above

Initial BMI was 44.0 ± 5.5 kg/m^2^ among the FA and 45.0 ± 5.3 kg/m^2^ among the IFA. Twelve years after surgery, BMI was 33.9 ± 6.4 kg/m^2^ in the FA and 36.5 ± 7.3 kg/m^2^ in the IFA. The %TWL was 23.2 ± 11.7% in the FA group vs 19.5 ± 12.6% in the IFA group (Fig. 2a). Furthermore, the %EWL in the FA was 55.7 ± 28.9% vs 46.0 ± 31.2% in the IFA (all p-values < 0.05) (Fig. 2b). WR was 32.1 ± 32.8% in the FA vs 38.4 ± 40.0% in the IFA (*p* = 0.052). WR > 20% was found in 191/292 (65.4%) FA and 148/205 (72.2%) of the IFA. Participant’s characteristics are shown in Table 1. Of the 205 participants in the IFA group, 105 (51.2%) participants did not attend at all. The FA were older than the 105 participants not attending at all, 40.8 (SD 8.9) vs 37.7 (8.4) years, and the FA had a higher %TWL than those not attending at all, 23.2 ± 11.7% vs 20.5 ± 12.3% (all *p*-values < 0.05). Between the groups, there was no difference in %WR 32.1 ± 32.8% vs 35.0 ± 38.6% (*p* = 0.445).

**Table 1:** Participants characteristics

*N* = 497	FA *N* = 292 (58.8%)	IFA *N* = 205 (41.2%)	*p*-value
Sex F/M	241/51	154/51	
Age at baseline (SD) years	40.8 (8.9)	38.4 (8.6)	*p* = 0.003
Mean follow-up time (SD) months	138.3 (18.0)	144.3 (20.1)	*p* < 0.001
BMI baseline kg/m^2^ (SD)	44.0 (5.5)	45.0 (5.3)	*p* = 0.037
BMI at nadir kg/m^2^ (SD)	28.8 (4.2)	30.6 (5.0)	*p* < 0.001
BMI 10–15 years kg/m^2^ (SD)	33.9 (6.4)	36.5 (7.3)	*p* < 0.001
TWL % to nadir kg/m^2^ (SD)	34.1 (8.2)	31.8 (8.7)	*p* = 0.002
TWL % 10–15 years (SD)	23.2 (11.7)	19.5 (12.6)	*p* < 0.001
∆BMI baseline → 10–15 years (SD)	− 10.1 (5.5)	− 8.5 (5.7)	*p* < 0.002
EWL % (SD)	55.7 (28.9)	46.0 (31.2)	*p* < 0.001
≤ 50%EWL 10–15 years N (%)	126 (43.7%)	111 (54.4%)	
≥ 50%EWL 10–15 years N (%)	162 (56.3%)	93 (45.6%)	
WR % 10–15 years (SD)	32.1 (32.8)	38.4 (40.0)	*p* = 0.052
WR < 20% 10–15 years N (%)	101 (34.6%)	57 (27.8%)	
WR > 20% 10–15 years N (%)	191 (65.4%)	148 (72.2%)	
Level of education			
≤ 12 year of school	189/291 (38.1%)	144/205 (29.0%)	*p* = 0.216
> 12 year of school	102/291 (20.6%)	61/205 (12.3%)	
Level of income			
< 75,000 Euro	179/290 (36.2%)	145/205 (29.3%)	*p* = 0.038
> 75,000 Euro	111/290 (22.4%)	60/205 (12.1%)	
Physical active			
< 150 min/week	197/266 (42.9%)	142/193 (30.9%)	*p* = 0.907
> 150 min/week	69/266 (15.0%)	51/193 (11.1%)	
Multivitamin			
Compliant	227/292 (45.8%)	143/204 (28.8%)	*p* = 0.054
Non-compliant to multivitamin	65/292 (13.1%)	61/204 (12.3%)	
Self-rated health			
Excellent/very good/good	187/291 (37.8%)	119/204 (24.0%)	*p* = 0.181
Fair/poor	104/209 (21.0%)	85/204 (17.2%)	

*FA*, frequent attendees; *IFA*, infrequent attendees; *F*, female/*M*, men; *BMI*, body mass index; *TWL*, total weight loss; *EWL*, excess weight loss; *WR*, weight regain in % of maximal weight loss; nadir = lowest weight measured at 1–2 years after surgery; *∆BMI*, baseline BMI-BMI 10–15 years after surgery

**Fig. 3:**
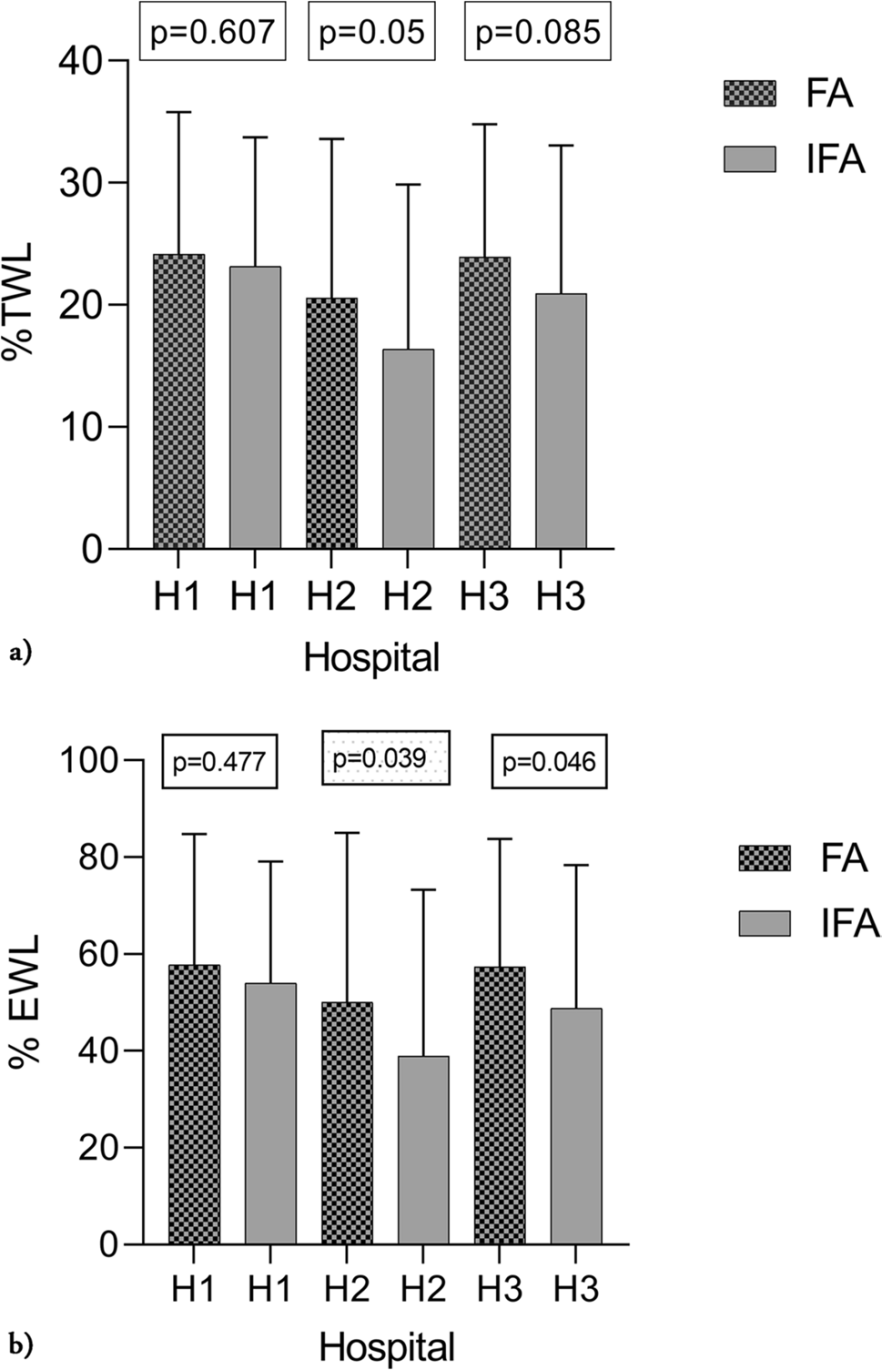
**a** %Total weight loss (%TWL) 12 years after surgery in frequent attendees (FA) and infrequent attendees (IFA) at the educational programs at each of the three hospitals. Results presented as mean ± SD, *p*-values between FA and IFA at each hospital in box above. **b** %Excess weight loss (%EWL) 12 years after surgery in frequent attendees (FA) and infrequent attendees (IFA) at the educational programs at each of the three hospitals. Results presented as mean ± SD, *p*-values between FA and IFA at each hospital in box above

Participants from hospital 1 and hospital 3 had higher %TWL and %EWL than hospital 2, see Fig. 3a and b. The comparison of categorical variables between the FA and IFA is summarized in Table 1. Regarding socioeconomic variables, more participants among the FA had a family income higher than 75.000 Euro/year than the IFA. There was no difference in physical activity between FA and IFA at the cut-off limit of 150 min/week, 69/266 vs 51/193 (*p* = 0.907).

From both groups (FA and IFA), there were 120/459 (26.1%) participants who reported physical activity of more than 150 min/week. Those who were active more than 150 min/week had higher %TWL and %EWL after 12 years, and WR was lower than in the 339 (73.9%) participants with activity < 150 min/week. TWL was 25.1 ± 10.5% vs 20.4 ± 12.4%, EWL was 60.5 ± 25.1% vs 48.4 ± 30.9%, and WR was 26.8 ± 29.3% vs 38.2 ± 37.1% (all *p*-values < 0.05). Fifty-four percent (245/451) of the participants reported high intensity at their work-out compared to 46% (206/451) with low intensity. Participants with high intensity had higher TWL 22.7 ± 11.5% vs 20.1 ± 12.7% and EWL 54.5 ± 28.4% vs 47.3 ± 31.2% (all *p*-values < 0.05) than those with lower intensity. Thirty-nine percent of the participants (175/451) had low intensity at work-out lasting less than 150 min/week.

Regarding participant’s self-rated health and their compliance to take recommended MVS, there was no difference between FA and IFA. Many of the participants made new long-lasting friendships in the education program, and about half of them suggested that the program could be improved by lasting longer and including more mental health issues. Sixty-four percent would like more individual consultations at the hospital’s outpatient clinic, even though 93% visited their GP annually or more often. The participants experiences and feedback regarding the education program is summarized in Table 2.

**Table 2:** Answers regarding the educational programs

Questions	Response n*	Yes	No
Felt support from other group members	387	315 (81.4%)	72 (18.6%)
Made new long-lasting friendships	382	172 (45.0%)	210 (55.0%)
Active member of the group	383	255 (66.6%)	128 (33.4%)
Afraid sensitive information would leak out when sharing information	384	51 (13.3%)	333 (86.7%)
I stopped coming as I felt no benefit of the program	384	58 (15.1%)	326 (84.9%)
Felt unsuccessful due to less weight loss than others	386	65 (16.8%)	321 (83.2%)
Would like more individual follow-up at hospital	483	309 (64.4%)	171 (35.6%)
GP once a year or more	448	229 (93.9%)	15 (6.1%)

^*^n: responses were different mostly due to those who did not attend the group sessions, they did not respond to these questions

## Discussion

The most novel findings in our study conclude that FA to the educational programs have improved weight loss compared to the IFA and tend to have less weight regain more than 10 years after surgery. In this retrospective multicenter cross-sectional study with a mean follow-up time of nearly 12 years after RYGB, almost 60% of the invited patients participated in the study and 59% of the participants classifies themselves as FA at the educational programs. Andreu et al. found that the number of attended support group sessions during the first-, second-, and fifth-year post-surgery predicted %TWL and %EWL at 1, 2, and 5 years [6]. Auge et al. recommend long-term multidisciplinary follow-up (four times during the first year and once or twice a year thereafter) to detect and prevent WR, nutritional deficiencies, and complications [15]. At our hospitals, the follow-up at the outpatient clinic was 2–5 years. In our study, the difference in BMI change between FA and IFA may seem small, but 1.6 BMI units correspond to approximately 5 kg which is clinically relevant and may have an impact on the participants mobility and the risk of developing comorbidities like diabetes, hypertension, or sleep apnea or not [16]. Furthermore, Whitlock et al. reported that a BMI increase of 5 kg/m^2^ gave 30% higher all-cause mortality and 40% higher cardiovascular disease mortality [17].

In our study, the FA had a higher age at baseline and higher weight loss than the IFA 12 years after surgery. Those who did not attend the education program at all have an even lower age at baseline and lower %TWL compared to the FA 12 years after surgery, but no difference in WR. Barka et al. also found a lower attendance rate among younger male patients [18]. The super-obese patients in Margo’s study were younger and had significantly higher WR compared to the older patients with morbid obesity [7].

After OS, daily physical activity is recommended to keep a lean body mass and improve insulin sensitivity, blood lipid profile, increase cardiovascular, and aerobic capacity [19]. Our results not only indicate that activity more than 150 min/week is advisable to keep the lost weight off and prevent WR in the long term but also indicate that intensity might replace duration and be more effective in terms of weight loss. In a newly published substudy from BAROBS with 50 participants, the group of weight regainers reports lower physical activity and have a higher intake of energy-dense foods [20]. Other short-term studies have found larger weight loss outcome for participants active more than 150 min/week [21, 22]. Participants usually overestimate physical activity, but the cut-off limit in our study is rather strict. Even if they overreport, it reveals an attitude towards physical activity. It is difficult for many participants to perform the recommended 150 min/week of exercise every week. However, educational programs give health professionals the opportunity to guide patients in overcoming challenges related to being physically active. Among our participants, some became active without participating in the programs.

Seventy-five percent of all participants reports taking MVS 12 years after surgery, and there is no difference between FA and IFA. Heusschen et al. reported a compliance rate of 55% at 12 months even though the participants received the MVS for free the first year after surgery [23].

We do not know why the educational programs did not appeal to the younger participants. But it might be as Luca et al. found that one-third of participants considered follow-up as unnecessary and a quarter referred to geographic distance, while only 7% stopped attending due to poorer weight loss [24]. Due to limited resources, all three programs had only two group sessions with psychologists. Half of the participants in our study suggested that the educational programs should focus more on mental health issues in the future.

We find no difference in self-rated health between FA and IFA. Support from the bariatric multidisciplinary team and other patients seems essential to optimize the long-term effects of obesity surgery and some patients may need more back-up as changing lifestyle is time-consuming and challenging. To help patients adopting WR strategies, it seems most reasonable to offer patients both follow-up at the outpatient clinic and group sessions lasting longer than until nadir weight is achieved. In the future, this may be done as a combination of digital and personal visits.

This study has limitations. Data regarding attendance to the programs are based on self-reporting. Furthermore, the data reported are based on educational programs in three hospitals with some structural difference such as curriculum, number of group sessions, and duration.

Our study’s strengths are a large sample size with 12 years follow-up where all participants have the same operation. The support groups started shortly after surgery and were still going on while the participants experienced WR which indicates our participants got early intervention. A relationship between the participants and healthcare professional was established prior to WR, which made it easier for participants to request guidance before the program ended.

## Conclusion

Participants attending an educational program lasting 2–3 years after RYGB have better weight loss outcomes 12 years later. Participants with the highest weight loss had a more comprehensive follow-up both individually and in groups. The educational programs lasted longer than the time to nadir weight which opened the possibility to help participants adopt WR strategies. There is no difference between the two groups regarding self-rated health, compliance to MVS, and physical activity. The most physically active participants from both groups have higher weight loss which confirms that a healthy lifestyle optimizes the effect of RYGB, but some WR seems unavoidable in the long term.

## References

[1] Cypess AM (2022). Reassessing human adipose tissue. N Engl J Med.

[2] Cambi MPC, Baretta GAP, Magro DO (2021). Multidisciplinary approach for weight regain-how to manage this challenging condition: an expert review. Obes Surg.

[3] O’Brien PE, Hindle A, Brennan L (2019). Long-term outcomes after bariatric surgery: a systematic review and meta-analysis of weight loss at 10 or more years for all bariatric procedures and a single-centre review of 20-year outcomes after adjustable gastric banding. Obes Surg.

[4] Courcoulas AP, King WC, Belle SH (2018). Seven-year weight trajectories and health outcomes in the longitudinal assessment of bariatric surgery (LABS) study. JAMA Surg.

[5] Mechanick JI, Apovian C, Brethauer S (2020). Clinical practice guidelines for the perioperative nutrition, metabolic, and nonsurgical support of patients undergoing bariatric procedures—2019 update: cosponsored by American Association of Clinical Endocrinologists/American College of Endocrinology, The Obesity Society, American Society for Metabolic and Bariatric Surgery, Obesity Medicine Association, and American Society of Anesthesiologists. Obesity (Silver Spring).

[6] Andreu A, Jimenez A, Vidal J (2020). Bariatric support groups predicts long-term weight loss. Obes Surg.

[7] Magro DO, Ueno M, Coelho-Neto JS (2018). Long-term weight loss outcomes after banded Roux-en-Y gastric bypass: a prospective 10-year follow-up study. Surg Obes Relat Dis.

[8] Nijland LMG, Reiber BMM, Monpellier VM (2022). The association between patient attendance to a perioperative group-based lifestyle program and weight loss after bariatric surgery. Surg Obes Relat Dis.

[9] WHO. Health literacy. 1998. Available from: https://www.who.int/activities/improving-health-literacy. Accessed 02 Feb 2022.

[10] Madan AK, Tichansky DS (2005). Patients postoperatively forget aspects of preoperative patient education. Obes Surg.

[11] Hindle A, de la Piedad GX, Brennan L (2017). Early post-operative psychosocial and weight predictors of later outcome in bariatric surgery: a systematic literature review. Obes Rev.

[12] Olbers T, Lönroth H, Fagevik-Olsén M (2003). Laparoscopic gastric bypass: development of technique, respiratory function, and long-term outcome. Obes Surg.

[13] Brethauer SA, Kim J, el Chaar M (2015). Standardized outcomes reporting in metabolic and bariatric surgery. Surg Obes Relat Dis.

[14] King WC, Hinerman AS, Belle SH (2018). Comparison of the performance of common measures of weight regain after bariatric surgery for association with clinical outcomes. JAMA.

[15] Auge M, Menahem B, Savey V et al. Long-term complications after gastric bypass and sleeve gastrectomy: what information to give to patients and practitioners, and why? J Visc Surg. 2022. 10.1016/j.jviscsurg.2022.02.004. 10.1016/j.jviscsurg.2022.02.004 35304081

[16] Miras AD, Kamocka A, Patel D (2018). Obesity surgery makes patients healthier and more functional: real world results from the United Kingdom National Bariatric Surgery Registry. Surg Obes Relat Dis.

[17] Whitlock G, Lewington S, Sherliker P (2009). Body-mass index and cause-specific mortality in 900 000 adults: collaborative analyses of 57 prospective studies. Lancet.

[18] Barka I, Sayedoff P, Garnier N (2021). Sociodemographic factors associated with loss to follow-up after bariatric surgery. Obes Surg.

[19] Tabesh MR, Maleklou F, Ejtehadi F (2019). Nutrition, physical activity, and prescription of supplements in pre- and post-bariatric surgery patients: a practical guideline. Obes Surg.

[20] Nymo S, Lundanes J, Aukan M (2022). Diet and physical activity are associated with suboptimal weight loss and weight regain 10–15 years after Roux-en-Y gastric bypass: a cross-sectional study. Obes Res Clin Pract.

[21] Amundsen T, Strømmen M, Martins C (2017). Suboptimal weight loss and weight regain after gastric bypass surgery-postoperative status of energy intake, eating behavior, physical activity, and psychometrics. Obes Surg.

[22] Donnelly JE, Blair SN, Jakicic JM (2009). American College of Sports Medicine Position Stand. Appropriate physical activity intervention strategies for weight loss and prevention of weight regain for adults. Med Sci Sports Exerc.

[23] Heusschen L, Berendsen AAM, Cooiman MI (2021). Optimizing multivitamin supplementation for sleeve gastrectomy patients. Obes Surg.

[24] Luca P, Nicolas C, Marina V (2021). Where are my patients? Lost and found in bariatric surgery. Obes Surg.

